# Insights Into the Role of Mortalin in Alzheimer’s Disease, Parkinson’s Disease, and HIV-1-Associated Neurocognitive Disorders

**DOI:** 10.3389/fcell.2022.903031

**Published:** 2022-07-04

**Authors:** Priyanka, Pankaj Seth

**Affiliations:** ^1^ Department of Cellular and Molecular Neuroscience, National Brain Research Centre, Gurgaon, India; ^2^ Current Address—Synaptic and Developmental Plasticity Group, Neurobiology Laboratory, National Institute of Environmental Health Sciences, National Institutes of Health, Research Triangle Park, NC, United States

**Keywords:** mortalin, HIV-human immunodeficiency virus, Alzheimer’s disease, viral infection, Parkinson’s disease

## Abstract

Mortalin is a chaperone protein that regulates physiological functions of cells. Its multifactorial role allows cells to survive pathological conditions. Pharmacological, chemical, and siRNA-mediated downregulation of mortalin increases oxidative stress, mitochondrial dysfunction leading to unregulated inflammation. In addition to its well-characterized function in controlling oxidative stress, mitochondrial health, and maintaining physiological balance, recent evidence from human brain autopsies and cell culture–based studies suggests a critical role of mortalin in attenuating the damage seen in several neurodegenerative diseases. Overexpression of mortalin provides an important line of defense against accumulated proteins, inflammation, and neuronal loss, a key characteristic feature observed in neurodegeneration. Neurodegenerative diseases are a group of progressive disorders, sharing pathological features in Alzheimer’s disease, Parkinson’s disease, multiple sclerosis, and HIV-associated neurocognitive disorder. Aggregation of insoluble amyloid beta-proteins and neurofibrillary tangles in Alzheimer’s disease are among the leading cause of neuropathology in the brain. Parkinson’s disease is characterized by the degeneration of dopamine neurons in substantia nigra pars compacta. A substantial synaptic loss leading to cognitive decline is the hallmark of HIV-associated neurocognitive disorder (HAND). Brain autopsies and cell culture studies showed reduced expression of mortalin in Alzheimer’s, Parkinson’s, and HAND cases and deciphered the important role of mortalin in brain cells. Here, we discuss mortalin and its regulation and describe how neurotoxic conditions alter the expression of mortalin and modulate its functions. In addition, we also review the neuroprotective role of mortalin under neuropathological conditions. This knowledge showcases the importance of mortalin in diverse brain functions and offers new opportunities for the development of therapeutic targets that can modulate the expression of mortalin using chemical compounds.

## Introduction

Neurodegenerative diseases of the central nervous system (CNS) are highly debilitating illnesses which impose substantial health burden affecting more than one billion people worldwide. These are characterized by the progressive loss of neurons and physiological dysfunction of the brain cells (Gorman, 2008; Chi et al., 2018), especially in diseases such as Alzheimer’s, Parkinson’s, and HIV-induced neurodegeneration. Clinical studies suggest disturbances in cognition (dementia and motor function impairment) and anatomical alterations in the brain, supporting a common disease spectrum, at least with respect to these diseases. The prevalence and incidence of these diseases increase dramatically with age and are also influenced by environmental and genetic factors (Hou et al., 2019). Among various neurodegenerative diseases, Alzheimer’s and Parkinson’s disease share common pathological hallmarks. Currently, more than 45 million people are affected by Alzheimer’s disease (AD) which is expected to grow to over 100 million by 2050. AD typically progresses with age from mild semantic memory problems to higher-order cognitive dysfunctions. Several brain autopsy studies showed severe brain pathology. Accumulation of Lewy bodies, beta-amyloid aggregate formation, and neurofibrillary tangle (NFT) pathology is characteristic of AD, many of these are also observed in the postmortem AD brains. Among all, beta-amyloid cascade theory is one of the major causes of dementia, it is hypothesized that increased production of insoluble beta-amyloid leads to aggregation and collapsed ubiquitin machinery which manifests as plaque formation in the brain. Detailed mechanistic studies indicate that the accumulation of these proteins leads to severe dysfunction of various brain cells and ultimately results in neurodegeneration (Mungenast et al., 2016; Wildburger et al., 2018; Hardy and Selkoe, 2002; Alzheimer’s Disease, 2022).

Parkinson’s disease (PD) is another progressive neurodegenerative disease that develops between the age of 55 and 65 years and is characterized by rigidity, resting tremors, and bradykinesia. It accounts for 10 million cases across the world. Ten percent of patients carry a single gene mutation, and the remaining 90% of patients are sporadic with no family history (Klein and Westenberger, 2012). Substantial reduction in dopaminergic neurons in substantia nigra pars compacta (SNpc) is the most consistent postmortem finding in patient’s brain (Jankovic, 2008; Kouli et al., 2018; Greenland et al., 2018). Dopamine replacement through L-DOPA has proven to reduce the symptoms and effectively manage the disease (Obeso et al., 2017). Together, AD and PD are one of the greatest burdens of age-related disorders. A unifying feature of AD and PD is the abnormal accumulation of proteins, leading to neuronal loss and several pathophysiological changes in the brain. These features include, but are not limited to oxidative stress, mitochondrial dysfunction (increased mitochondrial fragmentation, altered mitochondrial membrane potential, and ATP dysregulation), increased inflammation, and neuronal death.

Neurotropic viruses have neural-invasive and neurovirulent properties that damage the central nervous system (CNS). A large number of viruses have been reported in neurological diseases as they cause severe damage to neuronal cells and activate astrocytes and microglia leading to severe pathological and behavioral alterations. Human immunodeficiency virus-1 (HIV-1) breaches the blood–brain barrier and causes neuronal dysfunction leading to cognitive dysfunctions called HIV-associated neurocognitive disorders (HAND). Approximately 37.7 million people are living with HIV worldwide with more than 42.6% develop HIV-associated neurocognitive dementia (Wang et al., 2020a). HIV-associated neurodegeneration is characterized by impaired neuropsychological test performance and functional impairment and the spectrum ranges from asymptomatic to mild neurocognitive impairment to more severe HIV-associated dementia. The predominate clinical features combine a variety of neurological problems including dementia and difficulty in performing daily tasks (Eggers et al., 2017). HIV and its proteins are known to induce key pathology through altering various physiological functions of brain cells.

Despite of the differential pathological causes, these diseases share common neuro-inflammatory hallmarks, such as increased oxidative stress, accumulation of misfolded proteins into insoluble aggregates, synaptic dysfunctions, apoptosis, mitopathy, and chronic inflammation (Abramov et al., 2020; Chen et al., 2012; Taoufik et al., 2018; Martinez-Vicente, 2017). Dysregulated tripartite connection and accumulation of misfolded proteins are some of the most common underlying causes of neuronal loss and neurodegeneration. In the past few decades, more mechanistic insights into the role of heat shock proteins (HSPs) in the brain cells (in both physiological and pathophysiological conditions) have been observed (Buccellato et al., 2007; Miller and Fort, 2018; Taha et al., 2019).

From a traditional viewpoint, HSPs are evolutionarily conserved proteins known for their pro-survival role (Dukay et al., 2019). These proteins have evolved to cope with the heat stress. Decades of research showed that HSPs are much more important in physiological and pathological functions than their characterized aspects. It is a well-established fact that HSPs are versatile and play an important role besides classical molecular chaperoning (Schopf et al., 2017). They are widely studied and associated with several neurodegenerative diseases. In recent times, HSPs gathered the due recognition for their role in regulating some of the key CNS functions. The way in which HSPs regulate the fundamental functions of brain cells has been intensively studied over the past few decades (San Gil et al., 2017; Miller and Fort, 2018; Walsh et al., 1997). HSPs play important homeostatic functions in CNS notably by regulating oxidative stress, acting as a chaperone in terms of clearing accumulated proteins, maintaining protein conformations, mitochondrial biogenesis, and neuronal survival and functions (Böttinger et al., 2015; Voos and Röttgers, 2002; Lackie et al., 2017; Sweeney et al., 2017). They function virtually at all stages of life and take active participation in regulating stressful conditions and guarding cells from a wide range of deleterious effect of neurotoxicants. There are also growing evidence of HSPs in regulating cell differentiation, neurite outgrowth, and neuronal differentiation through specific pathways.

Unlike other HSPs, interestingly, mortalin is a heat un-inducible protein (Londono et al., 2012). Mortalin is localized in mitochondria, nucleus, endoplasmic reticulum (ER), cytoplasm, and detected in blood as it could be circulated or released in blood (Ran et al., 2000; Jubran et al., 2017). The ramified localization allows mortalin to perform diverse tasks through its interaction with various proteins. This also resulted in different names of mortalin such as HSPA9, Grp75, PBP74, and mtHSP70 (Kaul et al., 2002; Flachbartová and Kovacech, 2013). Mortalin is a highly complex protein that executes a plethora of functions in healthy and disease conditions. This includes maintaining cellular homeostasis, providing metabolic support to the cells, chaperoning of protein which includes folding and refolding of proteins, removing aggregated or misfolded protein, and majorly it is studied in regulating mitochondrial biogenesis (Deocaris et al., 2008; Londono et al., 2012; Dores-Silva et al., 2015; Deocaris et al., 2006). S A Bruschi et al. (1994) originally described mortalin as a mitochondrial stress protein (Bruschi et al., 1993; Bruschi and Lindsay, 1994). During stress, mortalin gets phosphorylated in order to rescue or reduce the cellular stress (Londono et al., 2012). Considering these changes in the cell, mortalin is known as an anti-stress protein, that confers resistance against oxidative stress and apoptosis and participates in reducing neurotoxicity induced by cerebral ischemia, amyloid-β, and synucleinopathy like neurotoxicants (Xu et al., 2009; Qu et al., 2012; Deocaris et al., 2007; Qu et al., 2011). These observations motivated a series of studies examining the role of mortalin under stress in different cell types. During acute to chronic inflammatory period, several savior pathways get dysregulated leading to alteration in the fundamental properties of the cells (Chen et al., 2010; Multhoff et al., 2011; Flusberg and Sorger, 2015). Partial loss or chemical-mediated inhibition of mortalin showed similar inflammatory responses in various types of cells (Honrath et al., 2017; Chiasserini et al., 2011). Such observations led to the foundation of mortalin as a critical regulatory protein associated with cellular homeostasis. Various studies showed a wide range of functions of mortalin, interestingly, mortalin has been observed to play both protective and destructive roles in various cell types. Several studies have reported that the dual role of mortalin could be dependent upon a threshold level of this protein or on its interacting partners. Besides to the explicit role of mortalin in maintaining cellular homeostasis, recently mortalin has been linked with mitochondria which paved the way to the idea of the role of mortalin in mitochondria-related dysfunction and diseases (Jin et al., 2006; Starenki et al., 2019; Mazkereth et al., 2016; Qu et al., 2012). Accordingly, mitochondrial mortalin (mtHSP70/mortalin) have been implicated in several complex mitochondrial functions ranging from maintaining mitochondrial membrane potential to mitochondrial damage or mitophagy.

In this review, we discussed the key mechanistic roles of mortalin in neurodegenerative (AD and PD) and HIV infection, focusing on progress in understanding mortalin as a key aspect of the brain cells as well as in astrocyte–neuron interplay. In this context, we will illuminate recent advances covering the role of mitochondrial mortalin that is not only crucial for mitochondrial biogenesis but is also involved in inflammation induced in several neuropathies. In addition, we have also highlighted the need for in depth research on mortalin in neurodegeneration and viral infection. The review also offers insights into how mortalin can influence the disease progression by activating and binding with various proteins which may open a new avenue for therapeutic development.

## Expression of Mortalin and its Role in Alzheimer’s Disease

Alzheimer’s disease (AD) is a neurodegenerative disorder, accounting for up to 70% of worldwide cases of age-related disorders (Apostolova, 2016). Alzheimer’s disease is a progressive neurodegenerative disorder, characterized by memory loss (dementia), cognitive decline, and behavioral defects such as impaired motor system, speech, and visuospatial orientation (Nelson et al., 2009). AD is a growing health concern. Typically, AD is a complex interplay between genetic and environmental factors, which affects the brain cells and influences their functioning. Clinicopathologic studies found the accumulation of highly insoluble, densely packed filaments—β-amyloid plaques (Aβ), and neurofibrillary tangles (NFTs) in the brain of AD patients. They are considered as a major pathological hallmark of AD and are still relied upon for a pathological diagnosis (Wisniewski et al., 1990). Both these proteins are the major cause of synaptic and neuronal loss in AD patients and strongly correlate with cognitive deficit (Terry et al., 1991; DeKosky et al., 1996).

Various evidence and hypothesis link mitochondrial dysfunction to the pathogenesis of AD. The advent of fluorescence labeling technologies and biochemical assays enabled direct visualization of mitochondrial DNA and morphometry of electron micrographs revealed severe mitochondrial abnormalities in AD with increased oxidative stress in neurons with mitochondrial abnormality (Sayre et al., 1997; Montine et al., 1996; Smith et al., 1997; Xie et al., 2013). A series of reports suggest that the cytotoxic effect of elevated oxidative stress and mitochondrial damage on the neuronal health initiates the vicious cycle of inflammation resulting in neurodegeneration and behavioral changes.

HSP70 is the central component of the cellular machinery which acts as a quality controller in regulating cellular and molecular mechanisms (translocating many proteins in and out of the cells) and assisting a wide range of protein folding processes (helps in folding and maintaining the native conformation of proteins) (Ruan et al., 2017; Craig, 2018; Cyr and Neupert, 1996). HSP70 are considered as a biomarker for oxidative stress. In the hypoxia model, HSP70 plays a critical role in regulating and reducing the cytotoxic effects of stress. Accumulated and misfolded proteins in AD pathology are also known as chaperonopathy in which chaperones are abnormal in structure and function or their quantitative levels are dysregulated. These observations strongly indicate toward the altered HSP70 machinery. Consistently, many subsequent studies using immunoprecipitation and immunolabeling techniques showed C-terminus HSP70 interacting protein (CHIP) being involved in the metabolism of beta-amyloid precursor protein (βAPP) using the proteasome-dependent pathway (Kumar et al., 2007). In particular, HSP70 is reported in degrading the Tau and Aβ oligomers through the proteasome system (Kundel et al., 2018). Such studies indicate toward the key potential of HSP70 in AD pathology.

In addition to these aggregated proteins pathology, an APOE (apolipoprotein E) gene has been widely studied and associated with the increased risk of AD physiopathology. APOE is also known to disrupt the blood–brain barrier (BBB) integrity and induce severe neuropathological events in AD brain (Chernick et al., 2019). In humans, three alleles of the APOE gene are detected: APOE2, APOE3, and APOE4. They are involved in maintaining synaptic integrity, neurite outgrowth, and clearance of neurotoxicants from the CNS. A hippocampi APOE-knockout mice model showed reduced mortalin expression along with activated glial cells and elevated oxidative stress. The increased oxidative damage and neuronal apoptosis were correlated with the reduced expression of mortalin. A meta-analysis of clinical and autopsy-based studies from the various brain regions showed the increased expression of APOE4 allele (Farrer et al., 1997). Interestingly, a differential (1D and 2D) gel electrophoresis showed differential regulation of mortalin under the APOE-targeted mice model. Various studies showed that out of three APOE variants, APOE3 and APOE4 are highly prevalent among AD patients. Upon further examination, the proteomics analysis found four isoforms of mortalin in the hippocampus of APOE4 transgenic mice. Interestingly, out of four isoforms, particularly “d isoform of mortalin” was increased in AD patients. However, APOE4 transgenic mice showed lower expression of “d isoform of mortalin” in the hippocampus (Osorio et al., 2007). These observations indicate toward the differential regulation of mortalin in the AD hippocampal brain. However, how differential expression of mortalin influences the APOE gene remains to be elucidated.

Oxidative stress plays a major role in AD. There is strong evidence from postmortem and experimental analysis of increased oxidative stress and guided pathways are the major sources of inflammation in AD, which is associated with modulated mortalin levels. Both increased reactive oxygen species (ROS) and nitric oxide are implicated in Aβ-mediated neurotoxicity and disease progression. Increased oxidative stress–mediated inflammation is found to activate apoptotic signal–regulating kinase 1 (ASK1) and JNK which is critical for neuronal stability and its functions (Kadowaki et al., 2005; Song et al., 2014). Interestingly, ASK1 and JNK pathways are also studied in various AD models. The chronic expression of oxidative stress creates an imbalance between oxidant and antioxidant systems which overwhelms the intrinsic antioxidant defense. Growing evidence supports a principle of oxidative stress–mediated mitochondrial dysfunction in AD pathogenesis and it is a well-known fact that mitochondria are a main source of ROS, categorized as mtROS. Aβ treatment or mutant APPs induce mitochondrial fragmentation and increase neuronal dysfunction (Barsoum et al., 2006; Wang et al., 2008). From a cell-based functional screening, a siRNA library found mortalin a major regulator of mitochondrial machinery (Park et al., 2014). Upon chemical (MKT077) and genetic (siRNA) inhibition of mortalin increases oxidative stress and triggers mitochondrial damage, moreover, the induction of beta-amyloid treatment in these mortalin inhibited neurons synergistically increases the neurotoxicity and mitochondrial damage, however, upregulation of mortalin in beta-amyloid–treated neurons remarkably reduces the beta-amyloid–mediated cellular damage (Park et al., 2014). Taken together, such reports support the critical role of mortalin in mitochondrial function and neurodegenerative diseases.

Notably, reduced expression of mortalin has been widely correlated with increased oxidative stress and mitochondrial dysfunction, therefore, an altered level of mortalin could be an indicator of cytotoxicity, considerably, the chronic inhibition of mortalin might be playing a role in inducing neuropathology-like features (Park et al., 2014; Liu et al., 2017; Zhu et al., 2013a). Upon further examination, histopathological analysis showed the reduced expression of mortalin in the human brain autopsy section of AD patients and also in AD mice models (Osorio et al., 2007). Considering mtHSP70/mortalin, recent evidence revealed significant upregulation in Drp-1, one of the major sources of increased mitochondrial fragmentation in AD patients and animal models (Oliver and Reddy, 2019; Park et al., 2014). Mortalin has been consistently correlated with various proteins as its binding partner and they are considered as one of the contributing factors of mortalin versatility, however, immunoprecipitation analysis revealed no direct binding of mortalin with Drp-1 in AD models. These studies suggest that the inhibition of mortalin is not directly regulating the mitochondrial fission machinery, but mortalin may still contribute to mitochondrial dysfunction. The knockdown of mortalin induces failure in regulating oxidative stress and induces alteration in mitochondrial machinery and related genes resulting in mitochondrial dysfunction and fragmentation, which is widely observed in AD pathology (Zhu et al., 2013b; Park et al., 2014).

In AD pathophysiology, beta-amyloid accumulation in mitochondria and its interaction with various mitochondrial proteins facilitates the activation of mitochondrial permeability transition pore (mPTP) which results in the loss of mitochondrial membrane potential (Δ*Ψ*m) leading to altered energy production and ROS modulation (Manczak et al., 2006; Pagani and Eckert, 2011; Wang et al., 2020b; Picone et al., 2014). Overexpression of mortalin conferred the inhibition of mPTP activation against beta-amyloid suggesting that mortalin can act as a cyto-protector by suppressing common pathogenic mechanisms (Qu et al., 2011; Qu et al., 2012). According to various studies, we hypothesize that mortalin is not directly regulating the mPTP activation. As per pathway studies, beta-amyloid induces massive calcium influx which further activates calcium release from the endoplasmic reticulum (ER) creating an overload in the cytosolic calcium that leads to mPTP opening. Interestingly, another study reveals that mortalin can regulate the calcium influx from ER through its interaction with a mitochondrial protein-VDAC (Qu et al., 2012; Qu et al., 2011). Hence, overexpression of mortalin under the influence of beta-amyloid might be influencing the calcium influx and regulating the further downstream signaling. Hence, studying the calcium–ER–mortalin pathway would help in understanding the mechanisms responsible for calcium-mediated mitochondrial damage.

Since the discovery of mortalin, it has been established as a protein involved in cellular immortality, various overexpression of mortalin induces immortality in the cells (Wadhwa et al., 2006; Wadhwa et al., 1994; Wadhwa et al., 2004). Mortalin has been widely studied in the cancer cells due to highly enriched expression of mortalin in various cancer cells (Lu et al., 2011; Na et al., 2016). Unlike cancer, reduced expression of mortalin in normal cells under the influence of glucose deprivation activates cell death, however, overexpression of mortalin in these cells inhibits the Bax (pro-apoptotic protein) and reduces the cytochrome C release, one of the key mitochondrial proteins and a master regulator of cell death (Liu et al., 2005), (Qu et al., 2012). In the AD model, beta-amyloid is found to upregulate the Bax expression which further activates neuronal death signaling, whereas the overexpression of mortalin counteracts the Bax expression and increases the Bcl2 (anti-apoptotic) levels (Paradis et al., 1996; Qu et al., 2011). In spite of these exemplary studies, the mechanism of action of mortalin remains unclear, which raises various questions such as—is the basal expression of mortalin sufficient to reduce the neurotoxic effects on brain cells? How and what are the threshold levels of mortalin that can influence the cellular conditions? In addition, there are no reports on the mortalin and beta-amyloid plaques or NFT, whether the overexpression of mortalin can clear the beta-amyloid plaques or it is only attenuating the stress mediated by beta-amyloid. Hence, to understand the plethora of mortalin in AD, such questions need to be explored.

Considering the potential role of mortalin in beta-amyloid–mediated neuronal damage. It is strongly suggestive that modulating mortalin levels either chemically (natural and synthetic) or genetically in AD can prevent beta-amyloid–mediated damage and might reduce the neuronal loss and neurocognitive dysfunction. AD is also defined as proteinopathy, and reduced mortalin expression can also be an additive factor in beta-amyloid plaque formation, failure of ubiquitin machinery, and accumulation of proteins. Understanding the interplay of mortalin and accumulated proteins in AD remains to be elucidated.

## Neuropathology of Parkinson’s Disease and Functional Significance of Mortalin

Parkinson’s disease (PD) is the second most common neurodegenerative disease which manifests with aging and presents clinically with parkinsonism. The main features include motor symptoms, abnormal posture, bradykinesia, and resting tremor. In 1817, James Parkinson described the behavioral defects such as shaking palsy in patients which was named after Parkinson, by Jean-Martin Charcot (Parkinson, 2002). Despite the widespread pathology, not all cell types are equally susceptible. PD is typified by major dopaminergic neuronal loss in the substantia nigra pars compacta (SNpc), a major region regulates motor functions. Studies found that reduced dopamine levels in basal ganglia lead to a movement disorder. Other than these, various other brain regions such as locus coeruleus, dorsal raphe nuclei, and post-ganglionic sympathetic neurons were also found to be affected, which contributed to dementia and autonomic dysfunction.

After almost a century, in 1912, Fritz Heinrich Lewy identified aggregated proteins outside the substantia nigra region and named them as Lewy bodies (LBs) (Holdorff et al., 2013). Reduced dopaminergic neurons of SNpc and LB formation are the classical characteristic pathological hallmarks observed in the PD brains. LBs are the abnormal intracellular aggregates containing proteins, such as alpha-synuclein (αSyn) and ubiquitin (Dickson et al., 2009; Holdorff et al., 2013). More than 60% of SNpc neurons are lost before any symptoms occur. The aggregation of alpha-synuclein either in Lewy bodies (LB) or Lewy neuritis (LN) form is considered a diagnostic marker (Halliday and McCann, 2010). In view of having effective treatment and early diagnosis in the field of neurodegeneration, several advances have been made in the past three decades and as a result, Tau protein, α-synuclein, DJ-1, and beta-amyloid in cerebrospinal fluid (CSF) and blood have been identified as potential diagnostic biomarkers for PD (Hong et al., 2010; Parnetti et al., 2013). Alpha-synuclein and LB formation are considered as a central event in PD pathology and a major cause of neuronal loss. Neuronal loss induces chronic neuro-inflammation characterized by reactive oxygen radicals, cytokines, mitochondrial dysfunction, and severely dysregulated kinase signaling. From several clinical studies, these pathological events are considered likely to be a major factor in disease recurrence or progression. Several advances have been made in this direction, and from a histopathological analysis, profound accumulation of α-synuclein in the mitochondria of the postmortem PD brain has been constitutively reported (Devi et al., 2008). Mitochondrial dysfunction is proposed to be an integral player in PD progression. The PD model not only alters the mitochondrial energy production but also plays an important role in inducing mitophagy, a critical event in neurodegeneration.

Interestingly, mitochondrial complex 1 deficiency has been reported in the substantia nigra of PD patients (Schapira et al., 1989), and is widely associated with increased mitochondrial ROS production, oxidative stress, reduced mitochondrial membrane potential, and mitophagy, along with α-synuclein pathology observed in multiple PD models (Devi et al., 2008; Hsu et al., 2000; Ludtmann et al., 2018). There is indisputable evidence for Parkin, PTEN-induced kinase 1 (PINK1), and DJ-1 mutation in PD patients, and other genes also involved in mitochondrial functions are also closely linked to controlling mitochondrial quality and providing an important connection to neurodegeneration (Cookson, 2012; Bonnet et al., 2012; Savitt et al., 2006). A series of reports on these mutated genes evaluated in various PD models exposed their role in disease progression and set the stage to elucidate the molecular mechanisms and the associated regulatory pathways (Cherian and Divya, 2020; Repici and Giorgini, 2019). Multiple lines of evidence point toward the role of mitochondrial dysfunction and mediated stress in the pathogenesis of PD. In terms of evaluating mitochondrial dysfunction in PD, a biochemical study showed the interaction of mortalin, which is a mitochondrial heat shock protein 70 (HSP70) with key genes involved in Parkinson’s disease such as Parkin, PINK1, and DJ-1 (Ge et al., 2020). Knockdown of DJ-1 is sufficient to disrupt the integrity and functionality of mitochondria and showed reduced protective capacity against cell death (Larsen et al., 2011; Hao et al., 2010). Interestingly, in hematopoietic stem cells (HSCs), mortalin and DJ-1 are found to interact and maintain the HSC properties by regulating mitochondrial health and oxidative stress. In fact, the inhibition of mortalin augments the ROS production in HSCs (Tai-Nagara et al., 2014). Intriguingly, functional impairment and oxidative stress are one of the pathological hallmarks in PD. Supporting the role of mitochondrial mortalin in PD, a shotgun proteomics multidimensional protein identification technology (MudPIT) profiled mitochondrial proteins from PD patients and PD model (DAergic neurons treated with rotenone) and found reduced expression of mortalin. Reduced expression of mortalin in DAergic neurons treated with rotenone induces oxidative stress and mitochondrial dysfunction (Jin et al., 2006). Overexpression and inhibition of mortalin play an important role in PD pathology. Considering these reports, a cohort study in a Spanish population found three variants of the mortalin gene (two missense-R126W and P509S and a 17 kb insertion in intron 8) in PD patients and the German cohort found a mutation in mortalin gene-A476T (De Mena et al., 2009; Burbulla et al., 2010). Cells carrying these variants showed increased proteolytic and oxidative stress, indicating a potential role of mortalin in inducing functional changes in the brain. In-depth consideration of mortalin in mitochondria with respect to PD, reduced expression of mortalin in PD is linked with dysregulated mitochondrial function, which was rescued by the overexpression of wild type mortalin (Jin et al., 2006).

Defects in several cellular systems have been implicated in the early onset of PD which triggers neuronal death. Reduced expression of mortalin-mediated cellular damage can be conceptualized as a relatively early event in neuronal death, through increased oxidative stress which likely participates in the recruitment of cell death activators. For the evidence of mortalin in the early onset of PD (EOPD), a cohort study screened 139 EOPD patients and detected one missense mutation in mortalin (p.L358P) which was not present in 279 control individuals. However, no changes were detected in mitochondrial morphology or related respiratory functions in EOPD patients (Freimann et al., 2013). Further supporting a role of mortalin in PD, a quantitative analysis showed a reduced cytosolic fraction of mortalin in the frontal cortex and in astrocytes of PD patients (Cook et al., 2016). Loss of mortalin in the *Drosophila* model mimics the PD pathology-like phenotype such as reduced ATP levels, abnormal locomotion, and posture defects (Zhu et al., 2013a). Dopaminergic neurons (DAs) are more sensitive to mortalin loss as compared to non-neuronal cells. α-synuclein treatment in DA neurons showed a reduction in mortalin expression, correlated with increased mitochondrial dysfunction and apoptosis (Basso et al., 2020; De Miranda et al., 2020). Interestingly, elevated levels of mortalin are found to regulate the acute toxicity, but chronic inflammation levels of mortalin are found to be consistently reduced. A serum-based study of PD patients and control individuals negatively correlated the levels of mortalin to α-synuclein levels. Levels of mortalin were lower in PD patients whereas α-synuclein was elevated. Taking reference from these studies, mortalin has been proposed to be used as a potential marker for PD diagnosis (Singh et al., 2018).

In general, the cellular systems have extensively evolved to deal with abnormally folded and aggerated proteins such as α-Syn. In this mechanism, mortalin is one of the key players in the ubiquitin–proteasome system (UPS). PINK1 and Parkin are commonly studied in autosomal recessive PD. PINK1 encodes PTEN-induced serine/threonine kinase 1 and Parkin is encoded by PRKN- E3 ubiquitin ligase Parkin. PINK1/Parkin are one of the well-studied pathways in PD pathogenesis. Several findings firmly establish their role in mitochondrial quality control (MQC). Mitochondria in neurons face a unique challenge in Parkinson’s disease*.* In *in vitro* model systems such as Hela cells and DA neurons, knockdown of mortalin triggers mitochondrial dysfunction. Overexpression of Parkin notably rescues the mitochondrial dysfunction that was induced by the knockdown of mortalin (Yang et al., 2011). In *in vivo* and *ex vivo* studies, loss of mortalin causes intramitochondrial proteolytic stress which further increases the expression of HSP60, a marker of mitochondrial Unfolded Protein Response (mtUPR). The mtUPR pathway has been widely studied in a *C. elegans* model of PD. Rotenone, MPP+ (complex I inhibitor), and paraquat (ROS inducer) are known to induce mtUPR and used to chemically induce the parkinsonian model. Importantly, rotenone-induced mtUPR signaling was found to be reduced by the mortalin overexpression (Jin et al., 2006). Chemical inhibition of mortalin in neurons induces proteolytic stress, reminiscent of an α-Syn induced effect. Interestingly, mitochondrial dysfunction induced by the knockdown of mortalin was rescued by the overexpression of Parkin and PINK1 (Burbulla et al., 2014).

Taken together, several conclusions can be drawn: 1) severe mitochondrial dysfunction is reported in the PD model and PD patients; 2) mitochondria-mediated damage plays an important role in inducing neuro-inflammation; 3) mortalin and/or mortalin interacting proteins appear to be affected in PD; 4) mortalin can attenuate the Lewy bodies and α-Syn-induced neuronal toxicity; 5) Parkin and PINK1 can act as mitochondrial protector. These concepts have implications for the development of therapeutics aimed at LBs and α-Syn. Another approach may be to use endogenous activation of mortalin or pharmacological overexpression to reduce oxidative damage and clearance of the aggregated protein.

## Differential Role of Mortalin in HIV-1 Neuropathogenesis

Human immunodeficiency virus (HIV) is the causative agent of acquired immunodeficiency syndrome (AIDS). Although it is an immune system diseases as it targets CD4 positive cells, HIV also infects the CNS *via* a “Trojan horse” model—invading the CNS by breaching the blood–brain barrier (Anderson et al., 2010; Izquierdo-Useros et al., 2010). Once it gains access to brain tissue, HIV-1 target microglial cells, as these cells allow multiplication of HIV-1 and infects other glial cells particularly astrocytes. Several reports found severe neuropathological conditions in HIV/AIDS patients such as memory loss, and dysregulated cognitive and motor functions (Glass et al., 1993; Marino et al., 2020). HIV-1-associated neurocognitive disorders (HAND) represent a range of neurological dysfunction similar to other neurodegenerative diseases such as Alzheimer’s disease (Putatunda et al., 2019). Despite the immense success of combinatorial antiretroviral therapy (cART), various viral proteins are being detected in the cerebrospinal fluid (CSF) and the autopsy brain sections of infected patients (Kaiser et al., 1989; de Almeida et al., 2022). HIV-1 viral proteins (Nef, Vpr, and Tat-transactivator of transcription) have been highlighted in contributing to the ample neuronal loss (Darbinian et al., 2020; Wang et al., 2017; Cheng et al., 2007; Sami Saribas et al., 2017). Among these, HIV-1 Tat and gp120 are highly neurotoxic thereby contributing to the neuronal loss in HIV/AIDS patients.

HSPs have been shown to play an important role in the life cycle of various viruses (Iyer et al., 2021; Gao et al., 2014). Their critical role has been demonstrated in HIV-1 neuropathogenesis either through their interaction with viral proteins and promoting viral replication or providing aid in inducing viral toxicity (Iyer et al., 2021; Pan et al., 2018; Brenner and Wainberg, 2001). Previously, HSP40, co-chaperone of HSP70 was found to interact with the HIV-1 negative regulatory factor (Nef), and this interaction is crucial for Nef-mediated toxicity and found to be involved in viral gene expression and replication (Kumar et al., 2011). The overexpression of HSP70 in several cell lines showed reduced Vpr replication, rescue of the cell cycle arrest, and increased cellular proliferation (Iordanskiy et al., 2004a; Iordanskiy et al., 2004b). HSPs are widely known to interact with several protein partners, which facilitate their multifaceted functions. Co-immunoprecipitation analysis showed direct interaction of Vpr-HSP70 (Iordanskiy et al., 2004a). However, several HSPs and viral protein interactions are known to facilitate viral replication and viral protein formation (Kim and Oglesbee, 2012; Lubkowska et al., 2021; Wan et al., 2020). Perhaps, there might be a further classification of the distinct HSPs which helps in promoting viral replication or others that might play a defensive role against virus production, or it could be possible that same HSPs has different effects under distinct conditions. However, this observation indicates a protective role of HSP70 against HIV. Further efforts are needed to integrate the various proximal pathophysiological mechanisms that have been implicated through HSPs in HIV. For instance, does any particular HSP contribute in reducing HIV-mediated stress or vice versa? Do the stressful events reinforce the expression of HSPs? Due to the wide range of inter-connectedness of HSPs, few HSPs may be an initiating factor of neurodegeneration in HIV. More research is needed to clarify the mechanism of HSPs in viral replication.

Numerous *in vitro* and *in vivo* studies have found secreted and circulated Nef in the CSF of infected patients (Valcour et al., 2012; McPhee et al., 1998). Nef is an accessory protein of the human immunodeficiency virus (HIV) (Basmaciogullari and Pizzato, 2014; Olivetta et al., 2016; Jacob et al., 2021). It counteracts host defense through secretory pathways. It is found to be expressed in astrocytes in HIV and has been suggested to play an important role in the pathogenesis of HAND (van Marle et al., 2004; Chompre et al., 2013; Rivera et al., 2019). From an *in vivo* study, Nef is found to be released in extracellular vesicles and linked with increased HSP70 levels. This suggests a possible role of HSP70 in extracellular vesicles which leads to the release of Nef in astrocytes (Sami Saribas et al., 2017). This could be a potential mechanism for host–virus interactions and viral infection in neighboring cells through the exploitation of the astrocyte functions (Ali et al., 2010; McNamara et al., 2018). A membrane-based study identified various proteins anchored Nef which are required to facilitate Nef binding and secretion, this human brain cDNA library found mortalin as one of the potential interacting proteins with Nef (Kammula et al., 2012). Predominate expression of extracellular Nef has been widely reported in various HIV models and has been linked with deleterious effects. The extracellular release of HSPs in the extracellular milieu is detected in various viral infection and disease conditions (Multhoff, 2007; Kotsiopriftis et al., 2005). Experimental evidence showed “secretion modification region (SMR)” is highly conserved among all HIV-1 clades and is an essential domain for the Nef secretion. Recently, an *in silico* study found SMR forms a putative binding pocket which assists Nef in exosome release (ex-Nef). Further analysis showed that mortalin is one of the proteins which binds to SMR-pocket and facilitates ex-Nef secretion (Shelton et al., 2012). Previously, SMRwt peptides have been used to antagonize the effect of mortalin on cancer cells and effectively reduce the complement-dependent cell toxicity (Huang et al., 2019; Fishelson and Kirschfink, 2019). The trio of SMR–Nef–mortalin (interaction) is one of the factors known for severe infection in uninfected cells and increased inflammation. This could represent a novel mechanism whereby mortalin can influence the Nef release in cells. Upon verification of mortalin and SMR specification, SMR mutation appears to cause alteration in the SMR-Nef binding domain by reducing the binding affinity and interfere in ex-Nef secretion. Mortalin is likely responsible for the extracellular release of Nef which results in neuronal death. Consistent with this, a fluorescence-based study found reduced ex-Nef secretion in mortalin knockdown cells. Conversely, the overexpression of mortalin in these Jurkat cells positively elevates the ex-Nef levels in the supernatants (Shelton et al., 2012). These findings displayed the role of mortalin in the trafficking of viral and host proteins. However, the in-depth mechanism behind mortalin and ex-Nef secretion remains unclear. Other than mortalin, three proteins were also found to be interacting with Nef in the same study. The effect of these proteins in ex-Nef remains unclear. In order to find the mechanism of mortalin in Nef-induced pathogenesis, a clear understanding of the mechanism of mortalin–Nef interaction is needed.

In this article, we review the evidence of mortalin in ex-Nef secretions. During recent years extracellular vesicles have been widely studied in various diseases and have been implicated in drug delivery and in basic biology to understand vesicular trafficking and signaling. In terms of potential therapeutics, a detailed study of mortalin in various viral conditions may be a more promising approach.

With regard to mortalin playing important role in HIV-1, our histopathological study showed reduced expression of mortalin in HIV-1 human autopsy brain sections. Similar downregulation was observed in the human fetal brain–derived astrocytes (Priyanka et al., 2020). Of note, mortalin was found playing a role in ex-Nef secretion, another HIV-1 viral protein. This creates disparities and raises a few questions; 1) what the expression level of mortalin in Nef condition is, as only the mortalin-Nef interaction was studied earlier. One possible explanation is that mortalin is involved in at least one stage of the viral life cycle such as secretion and/or production and after that, it has been degraded or reduced. In agreement with this, several reports documented the positive role of HSP70 during the early phase of viral replication and binding. During the initial phase of infection, a significant increase in various HSPs have been reported which are found to be reduced at the later stage of viral infection (Pujhari et al., 2019; Csillik et al., 1982). The basic function of mortalin could depend upon the cell type. So far, very few reports documented the role of mortalin in the brain and in neurodegeneration. There are few protective mechanisms that operate in the brain to tackle stress and mortalin is among them. Astrocytes under ischemic stress induce severe mitochondrial damage and oxidative stress which leads to inflammation, and the overexpression of mortalin in these ischemic astrocytes rescued them from oxidative stress and mitochondrial dysfunction (Voloboueva et al., 2008). Astrocytes are an integral part of CNS, a wide range of studies represent the critical role of astrocytes in regulating neuronal functions in both physiological and pathophysiological conditions (Liu et al., 2018; Siracusa et al., 2019). However, astrocytes are found to gain toxic functions and lose a neuroprotective role in various disease conditions, including HIV-1 Tat (Fan and He, 2016; Zhou et al., 2004).

Tat is an early gene product and critical for viral replication and has been associated with inducing apoptosis, oxidative stress, and chronic release of inflammatory toxicants (Agrawal et al., 2012; Tang et al., 2020). Interestingly, Tat protein is known to induce neuronal death via both direct and indirect mechanisms (Buscemi et al., 2007). Moreover, Tat infection alone in an animal model is enough to induce HAND pathology and behavioral defects. Although neurons are not infected by HIV-1, the major cause of neuronal death is *via* infected astrocytes and microglia. These infected cells release cytokines such as TNF-α and IL-1β, increase ROS and NO production in neurons which results in synaptic dysfunction, neurite retraction, and neuronal death, which is a leading cause of neuropathogenesis and neurodegeneration. In addition, Tat protein is secreted from infected cells and taken up by uninfected bystander cells (Chen et al., 2002). Exogenous Tat expression in astrocytic culture induces dysregulation in their fundamental functions such as dysregulated glutamate transporter causing excitotoxicity and mitochondrial dysfunction (reduced mitochondria size, altered ATP production, and increased mitochondrial fragmentation), which induces neuronal death (Lecoeur et al., 2012; Teodorof-Diedrich and Spector, 2020; Teodorof-Diedrich and Spector, 2018). Given the clear association of mortalin in these functions, it suggests a potential role of mortalin in HIV-1 Tat induced neuronal toxicity. It is, hence, important to emphasize the role of mortalin in astrocyte-mediated neuronal damage in HIV-1 Tat.

While the normal functions of mortalin in brain cells are still unexplored, it is clear that mortalin plays an important role in regulating varied stress, importantly in the mitochondrial context. The kind of mitochondrial dysfunction and morphological changes that were widely found in astrocyte and neuronal culture treated with Tat, was reminiscent of a similar degree of functional and morphological changes as reported in HIV/AIDS patients. A recent study from our laboratory showed that the overexpression of mortalin along with HIV-1 Tat rescued mitochondrial functions (examined by ATP production) and reduced mitochondrial fragmentation, which is a product of Tat inflammation (Priyanka et al., 2020). This study showed a protective effect of mortalin in astrocytes in HIV-1 Tat condition. Overexpression of mortalin was found to degrade the Tat protein in astrocyte culture, leading to the observed protective effects. It is noteworthy that many studies have demonstrated mortalin and various other HSP70 proteins to be involved with the ubiquitin machinery. A mechanistic study showed that co-chaperone carboxyl terminus HSP70/90 (CHIP), an E3 ubiquitin ligase binds with mortalin. In cancerous cells, degradation of proto-Dbl (dbl proto-oncogene product) was found to be promoted by GRP75/mortalin through the CHIP-mediated ubiquitin–proteasome pathway (Niu et al., 2018). The ubiquitin role of mortalin has also been studied in other neurodegenerative diseases (Li et al., 2005; Londono et al., 2012). Treatment of MG132 in transfected astrocytes (with mortalin and Tat) revealed mortalin-mediated Tat degradation *via* the ubiquitin–proteasome pathway (UPP) (Priyanka et al., 2020). This study also highlighted the ubiquitin role of mortalin. Astrocyte-mediated neuronal damage is a key characteristic feature of Tat, as documented in several studies. Astrocytes-mediated neuronal stress is a major contributing factor in HAND. Neurons exposed to infected astrocytes supernatant induce dendritic spines retraction, axonal disruption, dendritic pruning, mitochondrial dysfunction, and neuronal apoptosis (New et al., 1998; Perry et al., 2005). Interestingly, the indirect examination of neuronal health upon co-overexpression of mortalin and Tat condition showed intact neuronal morphology and reduced neuronal death (Priyanka et al., 2020). These evidence suggests that mortalin is neuroprotective in HAND and can play similar roles in other neurodegenerative diseases.

It is evident that further approaches could improve our understanding in this regard. However, the following questions need further attention—1) how does Tat downregulate mortalin levels in the brain? 2) Which event is occurring first, whether a) mortalin-mediated Tat degradation, or b) mortalin-mediated rescue as both are observed at several levels in astrocytes and in neurons. Considering the evidence, it is a fair assumption that both the events are happening simultaneously or in parallel.

## Conclusion and Future Perspectives

**FIGURE 1:**
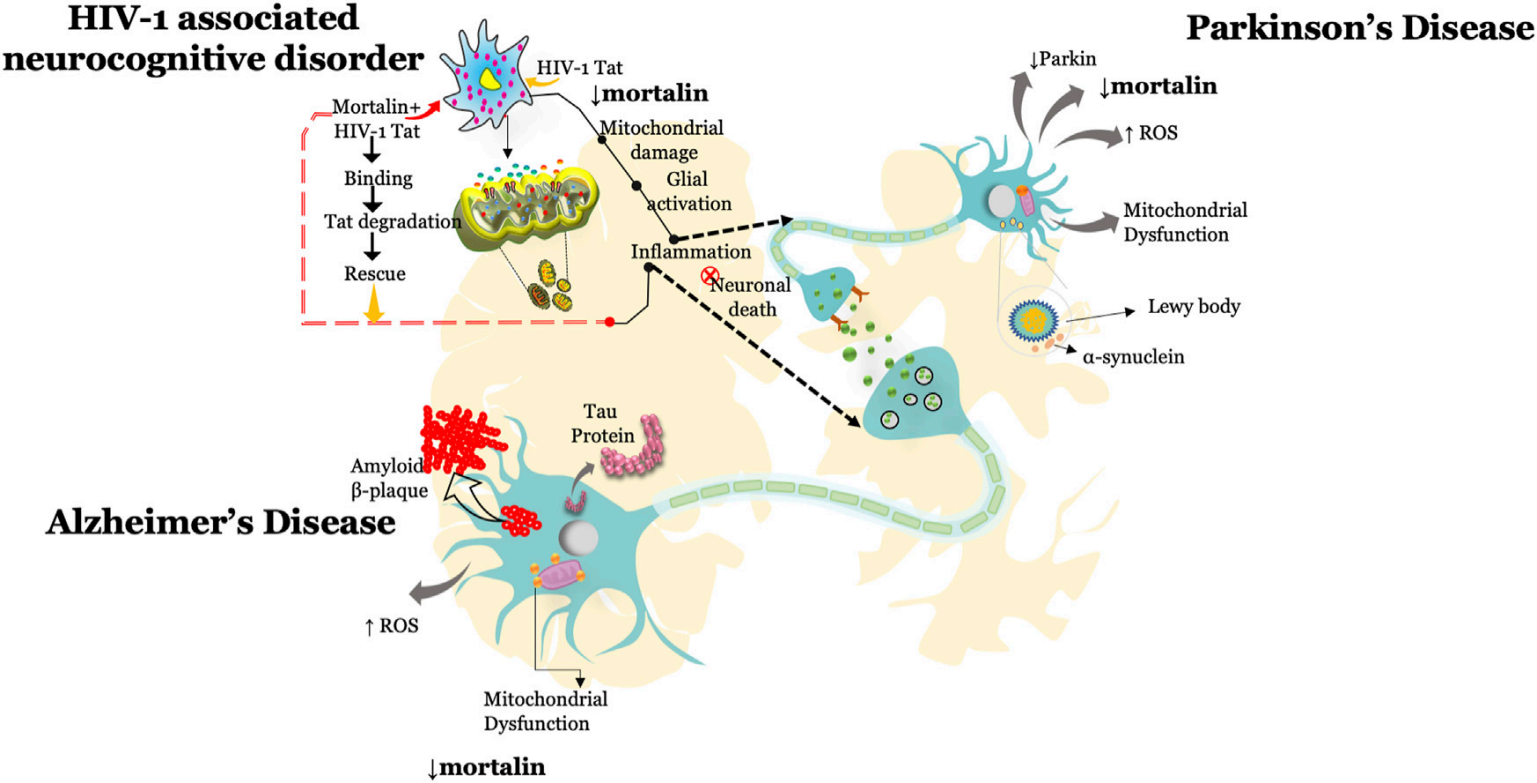
Tiff.

This review highlights the multiple roles of mortalin in the physiological and pathophysiological conditions associated with neurodegeneration and viral infection (Table 1). Several reports documented that knockdown of mortalin in normal cells resulted in increased oxidative stress, dysregulated mitochondrial functions and mitochondrial morphology, increased inflammation, and reduced cellular proliferation, whereas overexpression of mortalin increases the antistress property by reducing the ischemic stress and rescuing mitochondrial dysfunction. Such studies suggest the key role of mortalin in regulating physiological functions in neuronal and non-neuronal cells. Mortalin is positioned at the heart of mitochondria metabolism and serves as a key player in maintaining mitochondrial functions *via* distinct pathways and through its interaction with various proteins, or co-chaperones (Table 2). Here in this review, we highlighted the role of mortalin in the brain in AD, PD, and HAND (Figure 1). In current understanding, reduced expression of mortalin in the brain autopsy sections of AD, PD, and HIV-1 patients suggested its key role in brain cells. However, there is no clear understanding of how mortalin is reduced in these diseases. Various studies in this review highlighted the critical role of mortalin overexpression in attenuating beta-amyloid and alpha-synuclein toxicity in neuronal cells. Interaction of mortalin with various proteins like-Nef and Tat showed its pro-inflammatory and anti-inflammatory role, highlighting its bifunctional role. However, in depth research is warranted to understand these mechanisms better. We also noted that the overexpression of mortalin proved to be critical in reducing the detrimental effects of AD, PD, and HIV-1 on the brain cells. In what may seem like a paradox, mortalin is also essential for viral infection. Expression level of mortalin underpins the health of the cell; for example, excessive mortalin contributes to irregular cellular proliferation observed in cancer cells, whereas inhibition of mortalin promotes oxidative stress, increased mitochondrial functions, and reduced lifespan detected in neurodegenerative diseases such as Parkinson’s, Alzheimer’s, and HIV. Further efforts in elucidating the role of mortalin and its interacting partners is required to fully recognize and exploit mortalin’s protective role against the discussed disorders.

**TABLE 1:** Tabular overview of proteins modulated in Alzheimer’s disease, Parkinson’s disease, and HIV-1-associated neurocognitive disorder (HAND).

Disease	Specimen	Protein	Reference
Alzheimer’s disease (AD)	APOE-KO mice	Reduced mortalin	Osorio et al., 2007
	AD-patients-d-isoform of mortalin	Out of four isoforms of mortalin in AD- “d-isoform of mortalin” is upregulated	Osorio et al., 2007
	AD-brain autopsy sections	Reduced mortalin levels	Osorio et al., 2007
	AD patients and the mice model	Increased Drp-1 levels	Oliver and Reddy, 2019, Park et al., 2014
Parkinson’s disease (PD)	PD-autopsy brain sections	Reduced mortalin	Singh et al., 2018
	DAergic neurons treated with rotenone	Reduced mortalin	Jin et al., 2006
	Cohort study—Spanish population	Mutant mortalin-R126W and P509S	De Mena et al., 2009
	Cohort study— EOPD patients	Mutant mortalin-p.L358P	Freimann et al., 2013
	Cohort study—German population	Mutant mortalin-A476T	Burbulla et al., 2010
	PD astrocytes	Reduced mortalin	Cook et al., 2016
HIV-1- associated neurocognitive disorder (HAND)	Nef secretion	Mortalin facilitates Nef secretion	Shelton et al., 2012
	HIV-1 brain autopsy sections	Reduced mortalin	Priyanka et al., 2020

**TABLE 2:** Schematic representation of protein–protein interaction between mortalin and other proteins under different conditions.

Proteins interacts with mortalin	Experiment	Disease model and sample	Reference
Beta-amyloid precursor protein (βAPP)	Immunoprecipitation	AD-cultured skeletal myotubes, HEK	Kumar et al., 2007
Parkin, PINK1	Biochemical assays, immunoprecipitation	PD-skin biopsies, SH-SY5Y, HEK293 cells, and m5-7 MEF	Ge et al., 2020, L F burbulla et al., 2014
DJ-1	Immunoprecipitation	Hematopoietic stem cells (HSCs)	Tai-Nagara et al., 2014
Vpr	Co-immunoprecipitation	HIV-1- HEK 293T and HeLa cells	Iordanskiy, Zhao, Dubrovsky, et al., 2004
HIV-1 Tat	Co-immunoprecipitation	HIV-1-human fetal progenitor–derived astrocytes	Priyanka et al., 2020
HIV-1 Nef–SMR	Insilco-cDNA library and membrane study, co-immunoprecipitation	HIV-1- Cos-7, Jurkat CD4^+^ T-cell derived from T-cell leukemia cells, U373-MAGI-CXCR4CEM, and HP-1 cells	Kammula et al., 2012, M N Shelton et al., 2012
E3 ubiquitin ligase	1D gel electrophoresis and co-immunoprecipitation	SKOV-3, Cos-7, and 293T-cell lines	Niu et al., 2018
